# Effects of *Artemisia herba-alba* or olive leaf (*Olea europaea*) powder supplementation on growth performance, carcass yield, and blood biochemical parameters in broilers

**DOI:** 10.14202/vetworld.2018.1624-1629

**Published:** 2018-11-26

**Authors:** Asma Ait-Kaki, Mamadou Tandiang Diaw, Fikremariam Geda, Nassim Moula

**Affiliations:** 1 Department of Biology, Faculty of Sciences, M’Hamed Bougara University of Boumerdes, 3500 Boumerdes, Algeria; 2 Department of Animal Production, ENSA, Thies University, BP A296 Thies, Senegal; 3 Department of Animal Production, Fundamental and Applied Research for Animal and Health, Faculty of Veterinary Medicine, Liege University, B-4000 Liege, Belgium

**Keywords:** *Artemisia herba-alba*, broiler, growth, *Olea europaea*, serum parameters

## Abstract

**Aim:**

This study was aimed to evaluate the effects of *Artemisia herba-alba* (white wormwood) or olive leaf (*Olea europaea*) powder supplementation on growth performance, carcass yield, and serum biochemical parameters in broilers.

**Materials and Methods:**

The study was conducted from April to May 2017 in Chemini region, Northern Algeria. A total of 60 1-day-old Ross 308 male chicks were divided into three groups consisted of 10 chicks, in each of two replications. The chicks in Group 1 were fed with a standard commercial diet (SCD); Group 2 received the same SCD with 2% supplementation of *A. herba-alba* powder; and Group 3 received the same SCD with 2% supplementation of *O. europaea* powder. Growth performance was measured with body weights every 2 weeks, daily feed intake, feed conversion ratio (FCR), and carcass yield at the end of 42 days of rearing. Blood samples were collected to analyze serum glucose, cholesterol, triglycerides, urea, and total protein levels.

**Results:**

Results showed that, at 42 days of rearing, supplementation of *O. europaea* and *A. herba-alba* significantly increased (p<0.001) mean body weight (2230.10±26.38 g and 2117.42±26.38 g, respectively, vs. 2336.66±27.88 g in chicks of Group 1), but there was no significant difference (p≥0.05) among the three diets for FCR or percentage carcass yield. Among the serum biochemical parameters, glucose was significantly affected (p<0.01) by supplementation of olive leaf powder (1.90 g/L: Group 3), compared to the SCD (2.24 g/L: Group 1) or Artemisia powder (2.05 g/L: Group 2). Moreover, the supplementation of olive leaf powder in Group 3 broilers significantly affected (p<0.05) the serum cholesterol level (0.95 g/L), compared to the control diet (1.13 g/L). There was no significant difference (p≥0.05) for the other selected serum biochemical concentrations, namely triglycerides, urea, and total protein.

**Conclusion:**

The supplementation of Artemisia or olive leaf powder into the diet for broilers improved body weight by about 5% or 10%, respectively, at slaughter with moderate changes in blood biochemical parameters.

## Introduction

Intensive poultry production in Algeria depends on exotic chicken strains and on imported feed ingredients such as soybean, corn, and mineral-vitamin complexes, which weaken the national poultry sector. In fact, the international price fluctuations of these feed ingredients destabilize the national markets and favor informal activities that are less controlled by public authorities. This increases the use of uncontrolled veterinary drugs (antibiotics, anticoccidials, and vaccines) for preventive purposes or as growth factors.

In Algeria, poultry production is mainly concentrated in northern cities where several medicinal plants, for instance, *Urtica dioica* (Azeghdhouf in Kabyle), *Artemisia herba* (Ifsi in Kabyle), and *Olea europaea* (Azemmour in Kabyle) are widely identified and used in traditional medicine. On the basis of their medicinal and nutritional properties, it is necessary to evaluate their potential as ingredients in animal nutrition through their effects on performance and biochemical parameters.

*A. herba*, also known as white wormwood, was described by Belhattab *et al*. [1], as dwarf shrub, and characterized by a thymol scent. Mahmoodly [2] reported its significant use in African folk medicine. Indeed, Artemisia is known for its anti-inflammatory effects [3], hypocholesterolemia, and hypotriglyceridemia [4-7]. In folk medicine, in Kabyle region of Algeria, this grass is used to treat several symptoms such as digestive problems (diarrhea and stomach upset) and respiratory problems (bronchitis and cough). Furthermore, antihelminthic and antileishmanial effects of Artemisia were reported in several studies [4,8-10].

The olive tree (*O. europaea*) is a millenary tree grown on the northern and southern coastal zones of the Mediterranean Sea. Olives and olive oil are the main products of the olive tree; the leaves are used in cosmetics, animal feed, and traditional medicine. Several studies have reported the hypotensive [11-13], hypoglycemic [14,15], and anti-infectious [16,17] effects of olive leaves.

This study aimed to evaluate the effects of supplementation of *A. herba-alba* or olive leaf (*O. europaea*) powder on growth performance, carcass yield, and serum biochemical parameters in broilers in a private farm located in Chemini, Bejaia, Algeria.

## Materials and Methods

### Ethics approval

Due to the lack of animal ethics commission in Algeria, the authors followed the regulations applied in Liege University, Belgium.

### Preparation and chemical composition of Artemisia or olive leaf powder

For this study, the olive leaves were harvested during the olive harvesting period (December 2016-February 2017) in the region of Chemini (Bejaia wilaya), about 260 km east of Algiers, the capital of Algeria. After traditionally drying the leaves, the leaves were transformed to powder using a cereal mill. Another ingredient used as a supplementation, *A. herba-alba*, was obtained from Bouhamza and Akbou regions (wilaya of Bejaia). The same drying techniques were used for the two plants; the final products were analyzed at the Laboratory of Forage Analysis (Department of Agricultural and Environmental Analyses, Province of Liege, Belgium). The parameters measured were dry matter (DM), crude protein (CP), crude fiber (CF), crude ash (CA), and selected macro and micro minerals (Table-1).

**Table-1:** Dietary ingredients of SCD for starter, grower, and finisher period and nutrient composition of Artemisia or olive leaf powder.

Ingredients (%)	Starter	Grower	Finisher
Corn	62	64	70
Soybean meal	30	28	22
Bran	5	5	5
Monocalcium phosphate	2	2	2
Premix	1	1	1
Nutrient level			
Metabolizable energy (kcal/kg)	2912	2933	2995
CP (%)	20.45	19.75	17.66
Calculated nutrient composition			
Analytical composition (g/kg)	Artemisia powder	Olive leaf powder	
DM	895	816	
CP	103	74	
CF	296	187	
CA	126	77	
Macro minerals (g/kg)			
Calcium	10.6	18.1	
Phosphorus	3.3	1.4	
Potassium	35.5	7.6	
Sodium	0.4	0.2	
Magnesium	2.5	2.9	
Micro minerals (mg/kg)			
Copper	15.1	NM	
Iron	299.7	NM	
Manganese	87.8	NM	
Zinc	31.0	NM	

SCD=Standard commercial diet; DM=Dry matter; CP=Crude protein; CF=Crude fiber; CA=Crude ash; NM=Not measured

### Animals and housing

The experiment was carried out for a rearing period of 42 days in a private farm located in Chemini, Bejaia, Algeria. A total of 60, 1-day-old Ross 308 male chicks (commercial strain) were divided into three groups consisted of 10 chicks, in each of two replications. The chicks in Group 1 were fed with a standard commercial diet (SCD); Group 2 received the same SCD with 2% supplementation of *A. herba-alba* powder; and Group 3 received the same SCD with 2% supplementation of *O. europaea* powder. The chicks were housed under identical conditions and provided lighting 16 h/day using fluorescent lights controlled by timers until the end of the experiment.

### Growth performance and carcass yield

A progressive feed transition was made between each rearing phase (starter, grower, and finisher). The feed was given *ad libitum* to the three groups, and the chicks were monitored daily to weigh the amounts of feed distributed and refused in the feeders. The mortality rate was determined as the percentage of the number of chicks died relative to their number at the beginning of the rearing period. Animals were individually weighed every 2 weeks, during the 42 days of rearing. The feed conversion ratio (FCR) of each group was calculated at the end of the experiment as a ratio of the amount of feed ingested during the rearing period to total weight gain. Toward the end of the experiment, the chicks were starved for about 12 h and slaughtered. Then, the animals were manually scalded and plucked. 1 h after the slaughter, carcass weight was calculated by removing feathers, blood, head, tarsus, and organs; carcass yield was measured as the ratio of the carcass weight to live weight multiplied by 100 and reported as percentage carcass yield.

### Serum biochemical analysis

Pre-slaughter blood samples were taken from the wing vein on five subjects of each batch (10 samples/group). Blood serum samples were collected and stored at –20°C until analyzed for serum glucose, cholesterol, triglycerides, urea, and total protein, which were determined using a spectrophotometer (LKBNovastec) and commercially available kits (SPINREACT, SA, Spain). The serum concentrations of glucose, cholesterol, triglycerides, and total protein were determined using the methods such as glucose oxidase-peroxidase, cholesterol oxidase-peroxidase (PAP), glycerol phosphate-PAP enzyme, and biuret (colorimetric test), respectively.

### Statistical analysis

The statistical analysis was performed using the SAS software (GLM procedure, SAS Institute, Inc., Cary, NC., USA) [18]. Data were verified for the hypothesis of normality, and the live weights were analyzed with a GLM including the fixed effects of diet (Groups 1, 2, and 3), age (d0, d14, d28, and d42), replication (1 and 2), and the age × diet interaction. One-way analysis of variance was performed to compare the FCR of the three diets and to identify the diet effect on the results of serum concentrations. Results were expressed as means and standard error of means unless otherwise stated. p<0.05 was considered to be statistically significant.

## Results

### Artemisia or olive leaf powder analysis

The analytical composition of Artemisia or olive leaf powder is reported in Table-1. The results of the analysis indicated that CP, CF, and CA contents were higher in Artemisia compared with olive leaves. However, olive leaves were more concentrated in calcium (18.1 vs. 10.6 g/kg) than Artemisia whose composition in potassium was higher (35.5 vs. 7.6 g/kg).

### Growth performance and carcass yield

The growth performance and percentage carcass yield of the three groups of broilers are reported in Table-2. The results indicated that there was no significant effect of replication (p≥0.05) on the evolution of the weights with the age of the chicks. At the end of 42 days of the rearing period, supplementation of *O. europaea* significantly increased mean body weight (p<0.001; 2117.42 g, 2230.10 g, and 2336.66 g), but there was no significant difference (p≥0.05) among the three diets for FCR (1.79, 1.87, and 1.81) or percentage carcass yield (70.52, 71.84, and 70.21), respectively, in Groups 1, 2, and 3. Of course, the mean body weights at day 0 showed that there was no significant difference (p≥0.05) among the groups at the beginning of the experiment (Group 1: 39.86 g, Group 2: 38.82 g, and Group 3: 39.96 g). The average daily gain of Group 3 was the highest followed by Groups 2 and 1, respectively; daily feed intake was not significantly different between the diets (p≥0.05). Furthermore, at the end of the experiment, mortality rates were 5, 10, and 15% for the Groups 1, 2, and 3, respectively, with 1, 2, and 3 chicks died in that order.

**Table-2:** Effect of Artemisia or olive leaf powder on growth performance and carcass yield of broilers (mean, pooled SEM).

Age	Diets			Pooled SEM	p-value			
	
	Group 1	Group 2	Group 3		Replication	Age	Diet	Age×diet
Live weight (g)								
Day 0	39.86	38.82	39.96	25.70	NS	**	**	*
Day 14	273.71	339.67	347.15	26.62				
Day 28	950.95^a^	1019.13^b^	1095.47^b^	26.62				
Day 42	2117.42^a^	2230.10^b^	2336.66^c^	27.12				
ADG (g/day)								
Days 0-14	16.71	21.49	21.94		-	-	-	-
Days 14-28	48.37	48.53	53.45		-	-	-	-
Days 28-42	83.32	86.48	88.68		-	-	-	-
Days 0-42	49.47	52.17	54.68		-	-	-	-
Feed intake (days 0-42, g)	3790.18	4170.23	4229.35		-	-	-	-
FCR (days 0-42)	1.79	1.87	1.81		-	-	-	-
Mortality: Number (%)	1 (5)	2 (10)	3 (15)		-	-	-	-
Carcass								
Yield (%)	70.52	71.84	70.21	0.57	NS	-	NS	NS

Group 1=Control SCD; Group 2: SCD+2% *Artemisia herba-alba*; Group 3: SCD+2% *Olea europaea leaf*. Within a row, mean values with different letters were significantly different (p<0.05);

** =p<0.001;

* =p<0.01; NS=p≥0.05; SEM=Standard error of means; FI=Feed intake; FCR=Feed conversion ratio; ADG=Average daily gain; SCD=Standard commercial diet

### Serum biochemical analysis

The mean serum concentrations of glucose, cholesterol, triglycerides, urea, and total protein are reported in Table-3. The results showed that, at the end of 42 days of the rearing period, glucose was significantly affected (p<0.01) by supplementation of olive leaf powder (Group 3: 1.90 g/L), compared to the SCD (Group 1: 2.24 g/L) or Artemisia powder (Group 2: 2.05 g/L). Moreover, the supplementation of olive leaf powder in Group 3 broilers significantly affected the serum cholesterol level (p<0.05; Group 3: 0.95 g/L and Group 2: 1.03 g/L), compared to the control (Group 1: 1.13 g/L) diet. There was no significant difference (p≥0.05) among the groups regarding the other selected serum biochemical concentrations, namely triglycerides, urea, and total protein.

**Table-3:** Effect of Artemisia or olive leaf powder on selected serum biochemical parameters of broilers (mean, pooled SEM).

Parameters (g/L)	Diet group			Pooled SEM	p-value	
	
	Group 1	Group 2	Group 3		Replication	Diet group
Glucose	2.24^a^	2.05^a^	1.90^b^	0.06	*	**
Cholesterol	1.13^a^	1.03^ab^	0.95^b^	0.05	NS	NS
Triglycerides	0.73	0.69	0.68	0.04	NS	NS
Urea	0.032	0.033	0.036	0.002	NS	NS
Total protein	26.66	27.11	26.02	0.77	NS	NS

Group 1=Control SCD; Group 2=SCD+2% *Artemisia herba-alba*; Group 3=SCD+2% *Olea europaea leaf*; Within a row, mean values with different letters were significantly different (p<0.05);

** =p<0.01;

* =p<0.05; NS=p≥0.05; SEM=Standard error of means; SCD=Standard commercial diet

## Discussion

### Artemisia or olive leaf powder

*A. herba-alba* is a North African species belonging to Asteraceae family. It is very popular on the steppes and the highlands of Algeria. In the steppes, for main rangelands of nomadic sheep production in Algeria, Artemisia represents an important fodder resource. According to some testimonials, Artemisia is also used for therapeutic purposes for humans and animals in Kabyle region of Algeria. It is used to ease digestion and soothe abdominal and liver pains; in addition, it is used as a dewormed and against diabetes. Steppes of white wormwood (*A. herba-alba*) have been continued to be among the best steppe rangelands in the high plains of Algeria [19].

Like other olive-growing regions, in Kabyle (Algeria), farmers traditionally distribute olive branches to animals either at trough or in field. It is difficult to estimate the percentage reserved for this use because it varies considerably from country to country. Anyways, the *ad libitum* distribution to ruminants does not pose any particular problem, except for the limited nutritional value of this feed. The fresh leaves and twigs were distributed in Greece to sheep and goats at 6% of animal live weight as the only feed and up to 10%, in the case of rabbits [20]. However, Zoiopoulos [21] suggested that the optimum level would be at 2.5% of live weight for ruminants.

Muñoz *et al*. [22] studied *ad libitum* diets of dried olive leaves, supplemented with a barley and a dried fishmeal protein (230 g/lamb/day), distributed to lambs weighing 36 kg, and reported a weight gain of 77 g/day, compared to lambs received 40 g of the same feed supplemented with urea in Spain. However, the control group consumed alfalfa hay and 200 g of barley had an increase of 154 g/day over 90 days. Moreover, Alibes and Berge [23] recommended the use of dried olive leaves like that of poor forages with protein supplement, some easily fermentable energy input, and mixture of minerals.

From practical point of view, little effort has been made in this region. For instance, Nigh [24] reported that, in Crete at the Kolymbari Center, the olive leaves collected from the oil mill were distributed fresh (<2 days old) at a rate of 15 kg/day to Holstein cows. Zoiopoulos [21] noted that currently, the distribution level of these fresh leaves has reached 30 kg/day for two meals. Similar amounts are distributed as silage after the harvest period. Although precise controls have not been scientifically performed, the author indicated a positive effect on milk production. Fresh leaves are also sometimes distributed to sows.

### Chemical composition of Artemisia or olive leaf powder

Chemical composition of Artemisia or olive leaf powder is presented in Table-1, with higher levels of CP and CF in Artemisia. The CP and CA contents of olive leaves are 11.8% and 18.7% of DM, respectively. The CP level reported in this study is closer to that reported by Cayan and Erener [25] and similar to the result of Fall-Touré *et al*. [26] but with leaves of *Faidherbia albida*. However, the CA content is 8-folds higher than the value reported by Cayan and Erener [25]. The chemical compositions of olive leaves and twigs depend on many factors such as variety of olive trees, agroclimatic conditions, time of sample collection, and different treatments. According to Alibes and Berge [23], the DM of fresh leaves is around 50%, while that of dry leaves is around 90%. The CP level in dry or insulated leaves is low, ranging from 7% to 8%, but slightly higher for fresh leaves. The fat content in olive leaves is about 6%, which is higher than that in conventional forages. The CF content is moderately variable. The content of parietal constituents substantially increases with the proportion of wood, especially the lignocellulose content; the level of lignin appears stable: 18-19%.

*A. herba-alba* has a much lower percentage of fiber which allows prejudging its appearance (17-33%). The DM contains between 6% and 11% CP, which consists 72% of amino acids. Beta-carotene levels vary between 1.3 and 7 mg/kg depending on the season [27]. The values recorded in the present study for CF (29.6%) and total nitrogen content (16.5%) are closer to those reported by Fenardji *et al*. [27]. The energy value of Artemisia is very low in the winter (0.2-0.4 UF/kg DM), increases rapidly in the spring (0.92 UF/kg DM), and decreases in the summer (0.6 UF/kg DM). In the autumn, the rain in September causes a new period of growth, and the energy value rises again (0.8 UF/kg DM) [28]. These nutritional content values make the plant very important in sheep production in Algeria.

The proportions of CP and CA in the DM of *A. herba-alba* are 10.3% and 12.6%, respectively. The Artemisia CP level reported in this study is higher than those found in stems and roots of *Artemisia annua* L. by Jae *et al*. [29] but remains well below than values reported by the same authors in leaves and in the inflorescence of the same species. These variations in Artemisia nutrient content according to the authors and diverse varieties used could be explained by several parameters such as origins and methods of cultivation, period of harvest, technology used during the harvest period, and methods of drying leaves [30-32].

### Growth performance and carcass yield

After 42 days of the rearing period, the mean weights of the three feeding groups, namely Group 1 (control, SCD), Group 2 (SCD+2% *A. herba-alba*), and Group 3 (SCD+2% *O. europaea* leaves) were 2117.42, 2230.10, and 2336.66 g, respectively. The results indicated a better growth performance for the Group 3 chicks, which was >200 g compared to Group 1 and over 100 g compared to Group 2 chicks. According to Zahid *et al*. [33], the powder of *A. herba-alba* used with the SCD in Group 2 improves the health of poultry due to its antibacterial, antioxidant, and antifungal properties. Furthermore, Gholamrezaie *et al*. [34] demonstrated that the addition of Artemisia leaves in poultry feed has the potential to improve daily weight gain and FCR. In the present study, the daily weight gain was positively affected, but no significant influence had been observed in an FCR. In terms of growth performance, the results with Artemisia in the present study were better than those reported by Cayan and Erener [25], which was measured at the end of rearing chicks weighing about 1840 g. The differences could be explained by different climatic conditions in which the feeding experiments were carried out (hot and humid seasons).

The FCRs of the control group and the two other groups received 2% feed supplements of Artemisia or olive leaf were generally low although numerically reached 1.79, 1.87, and 1.81, respectively. The closer the FCR between three groups proves the efficiency of Artemisia or olive leaf powder in diet of chicks as a supplement. The FCR for the feed complemented with the olive leaf powder found in the present work was lower than that reported byCayan and Erener [25], which was = 2.05. However, it was consistent with those reported by Chaabna [35]. According to Zahid *et al*. [33], *A. herba-alba* significantly improves FCR, which could reinforce the theory of high feed efficiency for Artemisia or olive leaf powder.

### Serum biochemical parameters

The results of the selected serum biochemical parameters studied were closer between the different groups, and no statistical differences were observed (p>0.05), except for cholesterol and glucose, which were significantly lower in the Group 3 chicks, which fed on the diet supplemented with 2% of the olive leaf powder. Parsaei *et al*. [36] reported that incorporation of olive leaf into the chick diet significantly decreased the blood values of cholesterol and glucose. Laaboudi *et al*. [37] demonstrated the hypoglycemic effect of olive leaves to treat diabetic rats, an effect close to that of certain molecules used in diabetes treatments. This effect can be explained by the contents of phenolic compounds in olive leaves extracts, as previously demonstrated by Fki *et al*. [38]. Despite the many positive effects of supplementing Artemisia into animal feed, cited in the literature, the present work could not show any positive effects on serum biochemical parameters of chicks, compared to the control group.

## Conclusion

The present study demonstrated that the supplementation of Artemisia or olive leaf powder into the broiler diet improved body weight by about 5% or 10%, respectively, at slaughter with moderate changes in blood biochemical parameters. The present work can contribute to a strategy of reducing the use of conventional poultry feed ingredients, mainly corn and soybeans, which might reduce the importation and production costs of broilers in Algeria.

## Authors’ Contributions

AA, MTD, and NM have conceived and designed the study. AA and NM conducted the study in the laboratory. NM and FG analyzed the data and performed statistical analysis. AA, NM, and FG drafted and revised the manuscript. All authors read and approved the final manuscript.
